# Not out of Danger

**Published:** 1892-09-10

**Authors:** 


### The Hospital

An Institutional Journal of

Science, Medicine, Nursing, and Philanthropy.

*For Hospitals, Asylums, Infirmaries, Dispensaries, Medical Practitioners, Students, Nurses, and the Charitable Public.*

Vol. XII.—No. 311.] [Sept. 10, 1892.

### Not Out of Danger.

The cold weather of the last few days has made us all breathe a little more freely. Every hour gained without an outbreak of cholera carries us forward on the way to safety. But the state of Hamburg still utters in our ears warning notes of danger. Much as we may desire to avoid panic we cannot shut our eyes to the fact that the deaths from cholera in Hamburg now number several thousands, and of those who have been attacked more than one in three have died. A little comfort may be taken from the consideration that the weakly people, and the people who have had some chronic disease to contend with have probably constituted a considerable proportion of the victims, and sanitation, or rather insanitation, of the most stupid and disgraceful kind has been responsible for a good many more. But there need be no doubt that many men, women, and children are now numbered with the dead who but for this dreadful visitation would still have had the prospect of a long and healthy life before them. Looked at in any way we please, a severe epidemic of cholera is a calamity of the first magnitude.

The British public may, we think, congratulate itself upon the sanitary army which has risen in its defence. Both regulars and volunteers have vied with each other in promptly marching to the front, and in their preparations against emergencies. It is well that this should be recognized and admitted. Red tape has been conspicuous by its absence, and critics of the most cynical turn of mind have not yet felt it necessary to make reference to the Circumlocution Office. The Local Government Board and its officials at the various ports and docks have worked with ardour and intelligence, and not without heroism. They ought to have full credit for what they have done. It is decidedly among the probabilities that but for the prompt and intelligent activity of our port medical officers cholera would now have been ravaging London and other large towns as it has ravaged Hamburg.

The general public hardly knows what is voluntary and what is State-controlled among our sanitary resources. It would be well, however, to give this matter a little consideration at the present time. The hospitals and the various nursing organisations, for example, consist entirely of voluntary associations. The most important by far of all our defensive organisations, if an outbreak of cholera were actually to occur, are the metropolitan and provincial hospitals. But these hospitals, with their medical and nursing staffs, are under no State control, and receive no State pay. Those who control them and those who maintain them are private persons. Yet we have seen the hospitals one by one wheeling into line and together constituting a sanitary and defensive army corps of the most imposing dimensions and of the highest order of efficiency. We who know intimately the nature of the splendid services which this army corps is capable of rendering cannot but congratulate our fellow-countrymen on the presence of so many volunteer sanitary regiments of veteran and proved capacity. The last announcement made is that even some of the special hospitals, not to be outdone by the more imposing general institutions, are clearing their decks for action and setting apart wards and appointing trained officials for possible cholera emergencies. We think it not only probable, but certain, that if the worst came to the worst, the London hospitals would be able to place five or six thousand beds at the disposal of the public, and a full complement of experienced medical men and thoroughly trained nurses. It is not, of course, to be supposed that there are five or six thousand empty beds at the hospitals waiting to be occupied. But the hospital authorities are evidently so convinced of the imperious necessity of dealing promptly and scientifically with the cholera epidemic, that they would not hesitate to send their less urgent cases home half-cured in order to make room for the still more hardly entreated victims of the cholera fiend.

It had hardly dawned upon the medical mind how great a part nursing now plays in medical practice, until the last week or two of cholera scare. The public would not have been surprised to hear of medical men, both old and young, volunteering for special service. It is not, however, the doctors who have hoisted a conspicuous flag. They know themselves to be always ready, and they feel sure that the public are as well aware of their readiness as themselves. They merely, therefore, stand at attention and await orders. But this is not the kind of quiet policy which has commended itself to nurses. They feel eager for the fray, and they are determined that the public shall know of their eagerness. The various nursing societies represented by the Trained Nurses' Club and Institute, the British Nurses' Association, and the thousands of unattached nurses at the various hospitals, have expressed their readiness for immediate service with ardour and enthusiasm. This is entirely as it should be, and in the case of nurses, as in that of doctors, we congratulate the public and ourselves upon the presence in our midst of a truly noble army of loyal, devoted, and thoroughly trained workers.

Many competent physicians begin to hope that the pressure of danger is diminishing for the present. If that be so, we shall all be devoutly thankful. But the experience of past epidemics of cholera warns us to expect a return of danger ; and not one return only, but perhaps two or three. The cold weather that is approaching may keep the enemy at a distance. But if we should be so foolish as to suppose that because he is likely to be closed up in winter quarters for a time therefore he is defeated and destroyed, we shall make a mistake which may prove overwhelmingly fatal. London, be it remembered, has a population of five millions ; it possesses some of the filthiest slums in the world; it has a broad and deep river, which, in spite of recent improvements, is still at times little better than a broad and deep sewer. Parts of London are supplied with water which contains a dangerous proportion of poison. We have, in fact, miles of inflammable material which requires but the application of the cholera match to be raised to overwhelming conflagration. What are we to do then? Are we to sit down and fold our hands because the shadow of danger is past or passing? That would be little better than insanity. The autumn and winter must be devoted to thorough and universal scavenging; to the purification of homes, of drinking water, and of the circumjacent atmosphere! It cannot be supposed for a moment that our instructed and intelligent public services will think of abating their vigilance. What we have to fear is the apathy and folly of the multitude. Surely, with the spectacle of Hamburg before us, all of us, even down to the poorest ratepayer, will unite as the heart of one man to prevent so dreadful a calamity as London overwhelmed with cholera.

The scientific, practical, and morally admirable attitude of doctors and nurses during the last few weeks has been such as to fill the minds of all observant persons with sincere respect. England has other meritorious protectors as well as those who wear the Queen's uniform. The striking and effective example set by those who best understand the terrible nature of a cholera epidemic ought to produce in every class of the community a deep seriousness of mind, and a steadfast resolution to do everything that is possible for the cleansing of our towns and villages from filth, the purging of our drainage system, and the purifying of our atmosphere from every form of disease germ by which it may be polluted. Cleanliness is said on high authority to be next to godliness; but in the case of cholera cleanliness is nothing less than absolute salvation.

